# Efficient Production of Live Offspring from Mouse Oocytes Vitrified with a Novel Cryoprotective Agent, Carboxylated ε-poly-L-lysine

**DOI:** 10.1371/journal.pone.0083613

**Published:** 2013-12-23

**Authors:** Hitomi Watanabe, Natsuki Kohaya, Maki Kamoshita, Katsuyoshi Fujiwara, Kazuaki Matsumura, Suong-Hyu Hyon, Junya Ito, Naomi Kashiwazaki

**Affiliations:** 1 Laboratory of Animal Reproduction, School of Veterinary Medicine, Azabu University, Sagamihara, Kanagawa, Japan; 2 Graduate School of Veterinary Sciences, Azabu University, Sagamihara, Kanagawa, Japan; 3 Japan Advanced Institute of Science and Technology, Nomi, Ishikawa, Japan; 4 Center for Fiber and Textile Science, Kyoto Institute of Technology, Matsugasaki, Kyoto, Japan; Institute of Zoology, Chinese Academy of Sciences, China

## Abstract

In cryopreservation of mammalian germ cells, unfertilized oocytes are one of the most available stages because these cryopreserved oocytes can be used for assisted reproductive technologies, including in vitro fertilization (IVF) and intracytoplasmic sperm injection. However, it has been generally reported that the fertility and developmental ability of the oocytes are reduced by cryopreservation. Therefore further improvement will be required. Very recently, a new cryoprotective agent (CPA), called as carboxylated ε-poly-L-lysine (COOH-PLL), has been developed to reduce physical and physiological damage by cryopreservation in mammalian stem cells. However, it is unclear the effect of COOH-PLL on fertility and developmental ability of vitrified oocytes. In this study, we used COOH-PLL as a CPA with ethylene glycol (EG) for vitrification of mouse oocytes. Cumulus-oocyte complexes (COCs) were collected from ICR mice and then vitrified with Cryotop using different concentration of COOH-PLL and EG. A combined treatment with COOH-PLL and EG showed high survival rate (more than 90%) of vitrified-warmed COCs after in vitro fertilization. In addition, the fertility and developmental ability of COCs vitrified with E20P10 [EG 20% (v/v) and COOH-PLL 10% (w/v)] or E15P15 group (EG 15% and COOH-PLL 15%) were significantly higher than those with E10P20 (EG10% and COOH-PLL 20%) or P30 group (PLL30%). The vitrified COCs in E20P10 group developed to term at a high success rate (46.2%) and it was significantly higher than that in control (E30) group (34.8%). Our present study demonstrated for the first time that COOH-PLL is effective for vitrification of mouse oocytes.

## Introduction

Unfertilized oocytes are at one of the most available stages for cryopreservation in mammals because the cryopreserved oocytes can be used for assisted reproductive technologies including in vitro fertilization (IVF) and intracytoplasmic sperm injection. The oocytes are also available for somatic cell nuclear transfer to recipients. In several mammalian species, researchers have thus attempted to improve the cryopreservation of oocytes. In our previous study, we used a vitrification method and improved the cryopreservation of oocytes in mice. The vitrification method was first reported by Rall & Fahy [1]. The major advantage of the vitrification method is the elimination of the physiological damage caused by intracellular or extracellular ice crystal formation, and the reduction of chilling damage by shortening the exposure to suboptimal temperature [2].

The vitrification method is also simpler and quicker than the traditional method (i.e., the slow-freezing method) because the embryos are out of the incubator for less than a few minutes in the vitrification method, whereas in the slow-freezing method the equilibration alone takes more than 20 min [3]. Therefore, many researchers have aimed to improve vitrification method. Recently we found that oocytes vitrified in calcium-free and ethylene glycol (EG)-supplemented medium showed slightly higher developmental ability compared to those of oocytes vitrified with dimethyl sulfoxide (DMSO) alone or both EG- and DMSO-supplemented medium [4]. It has been acknowledged that EG would be the ideal cryoprotective agent (CPA) for oocyte and embryo vitrification [5] because the permeability of EG is higher than those of other CPAs [6] and EG seems to have less toxicity than other permeable CPAs [7], [8]. However, a study suggests current CPAs, even EG, have some issues [9], and most of these CPAs showed toxic effects on cell viability in a dose-dependent manner [10]. The development of CPAs with high efficiency and low toxicity is thus necessary.

Matsumura and Hyon [11] found that the use of carboxylated ε-poly-L-lysine (COOH-PLL) reduced the risks of damage by ice recrystallization during freezing and thawing with anti-freezing protein-like activities. They also demonstrated the successful cryopreservation of murine L929 cells and rat bone marrow mesenchymal stem cells [10] with the addition of COOH-PLL as a CPA into common freezing solution. Matsumura et al. [12] also reported that the addition of PLL into the freezing solution dramatically improved the vitrification of human induced pluripotent stem cells with high efficiency. These results suggested the possibility that COOH-PLL is a more suitable CPA even in oocyte cryopreservation compared to other CPAs which have been used to date. To the best of our knowledge, the cryopreservation of germ cells including oocytes using COOH-PLL has been not reported. In this study, we examined whether COOH-PLL is applicable for the vitrification of mouse oocytes.

## Materials and Methods

All chemicals and reagents were purchased from Sigma-Aldrich (St. Louis, MO, USA) unless otherwise stated. The study was approved by the ethical committee for vertebrate experiments at Azabu University (ID#197110325-1) [13].

### Animals

The mice used in this study were Crlj: ICR females (4–5 wks old) for the collection of metaphase II (MII) oocytes, and Crlj: BDF1 males (12–24 wks old) were used for the sperm collection as previously reported [4]. The mice were purchased from Charles River Laboratories Japan (Yokohama, Japan). Mature female ICR mice (12–14 wks old) were used as recipients of the embryo transfer. Vasectomized male ICR mice (20–30 wks old) were used to induce pseudopregnancies. The mice were housed in an environmentally controlled room with a 12-h dark/12-h light cycle at a temperature of 23±2°C and humidity of 55±5% with free access to a laboratory diet and filtered water.

### Oocyte collection

Cumulus oocyte complexes (COCs) at the metaphase-II stage were collected from the oviducts of ICR female mice (4–8 weeks) that were superovulated by an i.p. injection of 5 IU equine chorionic gonadotropin (eCG; Nippon Zenyaku Kogyo, Tokyo) followed by 5 IU human chorionic gonadotropin (hCG; Asuka Pharmaceutical Co., Tokyo) 48 h later. Fourteen hours after the second injection, the females were sacrificed and their oviductal ampullae were removed. The oviductal ampullae were placed in oil, and COCs were collected from the oviductal ampullae with calcium- and magnesium-free modified PB1 (PB1(−))[14].

### Evaluation of permeability of COOH-PLL

We previously reported the synthesis of the polymeric cryoprotectant PLL (0.65) [10]. To synthesize PLL (0.65), 25% (w/w) PLL aqueous solution (10 mL; JNC Corp., Tokyo, Japan) and succinic anhydride (1.3 g SA; Wako Pure Chem. Ind. Ltd., Osaka Japan) were mixed and reacted at 50°C for 1 h to convert 65% amino groups to carboxyl groups. To clarify the permeability of COOH-PLL as a CPA, we used fluorescein isothiocyanate (FITC)-labeled COOH-PLL. For fluorescent labeling, a 25% COOH-PLL solution was reacted with FITC at a 1/100 molar ratio to PLL for 6 h at 50°C. FITC–PLL was purified by dialysis (cutoff molecular weight: 10 kDa, Spectra/Por, Spectrum Laboratories, Inc., CA, USA) against water for 72 h. Reaction with SA was performed to obtain FITC-labeled COOH-PLLs. Oocytes were exposed to PB1(-) supplemented with FITC labeled COOH-PLL (5% (w/v)) for 5 min. After exposure, interaction of COOH-PLLs with oocytes was observed by a fluorescent microscope (Olympus IX-71, Tokyo, Japan) using the FITC-labeled COOH-PLLs.

### Vitrification of oocytes

Vitrification was performed using Cryotop, as reported [15] with some modifications. In brief, COCs were placed in equilibrium solution [15% (v/v) ethylene glycol (EG), and 20% (v/v) fetal calf serum (FCS) in PB1(−)] for 3 min and then transferred into vitrification solution [30% (v/v) EG, 20% (v/v) FCS, and 0.5 M sucrose in PB1(−)] for 1 min. To clarify the effect of COOH-PLL, different concentrations of CPA were used for the equilibration and vitrification solutions (Table 1). The COCs were placed on a sheet of Cryotop (Kitazato BioPharma Co., Shizuoka, Japan) in a small volume of the vitrification solution. The Cryotop was plunged into liquid nitrogen when the COCs were exposed to the vitrification solution for 1 min, and then stored for at least 1 wk. The COCs were warmed by immersing the Cryotop into a warming solution composed of 0.5 M sucrose + 20% FCS in PB1(−) at 37°C for 3 min, and then put with 20% FCS in PB1(−) at 37°C for 5 min. Survival of the vitrified-warmed oocytes was morphologically evaluated. After being washed three times with TYH [16], COCs were transferred into a 100-µL drop of TYH and then used for IVF.

**Table 1:** Cryoprotectants in equilibration and vitrification medium for mouse oocytes.

Treatment	Equilibration medium*	Vitrification medium#
Control (E30)	15%EG	30%EG
E20P10	10%EG + 5%PLL	20%EG + 10% PLL
E15P15	7.5%EG + 7.5% PLL	15%EG + 15% PLL
E10P20	5%EG + 10% PLL	10%EG + 20%PLL
P30	15% PLL	30%PLL

*These cryoprotectants were added into calcium free PB1 supplemented with 20% FCS.

^#^ These cryoprotectants were added into calcium free PB1 supplemented with 20% FCS and 0.5 M sucrose.

### In vitro fertilization

After dissections, epididymides were removed and placed in a 35-mm sterile plastic dish containing 400 µL R18S3 medium [17]. The epididymal sperm were counted with a hematocytometer, and sperm motility and viability were evaluated as reported [18]. Namely, the sperm motility was assessed visually and determined by direct observation at 37°C under light microscopy at 100×. For the cryopreservation, spermatozoa were loaded into 0.25-mL plastic straws (Fujihira Industry, Tokyo). The straws were exposed to liquid nitrogen (LN) vapor (about −150°C) for 10 min and then plunged into LN and stored for at least 1 wk. For thawing, the straws were kept in a 37°C water-bath for 10 sec and the contents were then expelled into a 35-mm sterile plastic dish.

The post-thaw sperm viability and motility were evaluated as described above. The frozen-thawed spermatozoa were resuspended by TYH medium, and the number and motility of the sperm were assessed as described above. Fresh and frozen-thawed spermatozoa were incubated for the induction of sperm capacitation in TYH for 2 h or 1 h, respectively. The sperm were then added into the TYH drops containing COCs (final sperm concentration was 2×10^6^ sperm/ml) and were co-cultured for 6 h. After culture, COCs were transferred into a 100-µL drop of KSOM-AA [19] supplemented with 0.1% hyaluronidase.

The cumulus cells of COCs were then removed by gentle pipetting. The oocytes were washed three times in KSOM-AA and then evaluated for fertility using an inverted phase-contrast microscope (Olympus, Yokohama, Japan). At 6 h, Oocytes with two pronuclei were considered fertilized. Only fertilized oocytes were transferred into 100 µL of KSOM-AA and were cultured up to 120 h at 37.5°C under 5% CO_2_ in air. Cleavage and blastocyst formation of the oocytes were evaluated at 18 h and 114 h post-fertilization, respectively.

### Embryo transfer

To evaluate in vivo development of the IVF oocytes, putative embryos were incubated for 18–21 h after IVF and then transferred into the oviducts of recipients after the induction of pseudopregnancy as described [4]. Female mice as recipients for embryo transfer were mated with vasectomized males on day 0 between 16:00 and 22:00 to induce pseudopregnancies. On day 2 between 9:00 and 12:00, nine to ten embryos at the 2-cell stage were transferred into each oviduct of the recipients. On the morning of day 21, pups derived from the transferred females were checked and the normal phenotype of the offspring was also confirmed.

### Statistical analyses

Each experiment had at least three replicates. More than 100 oocytes were used for each treatment group in this study except for the embryo transfer. All percentage data were subjected to arcsine transformation before the statistical analysis. The data were analyzed by one-way analysis of variance (ANOVA) and Tukey's test. *P*<0.05 was considered significant. Data are shown as means ± standard error of means (S.E.M.).

## Results

**Figure 1:**
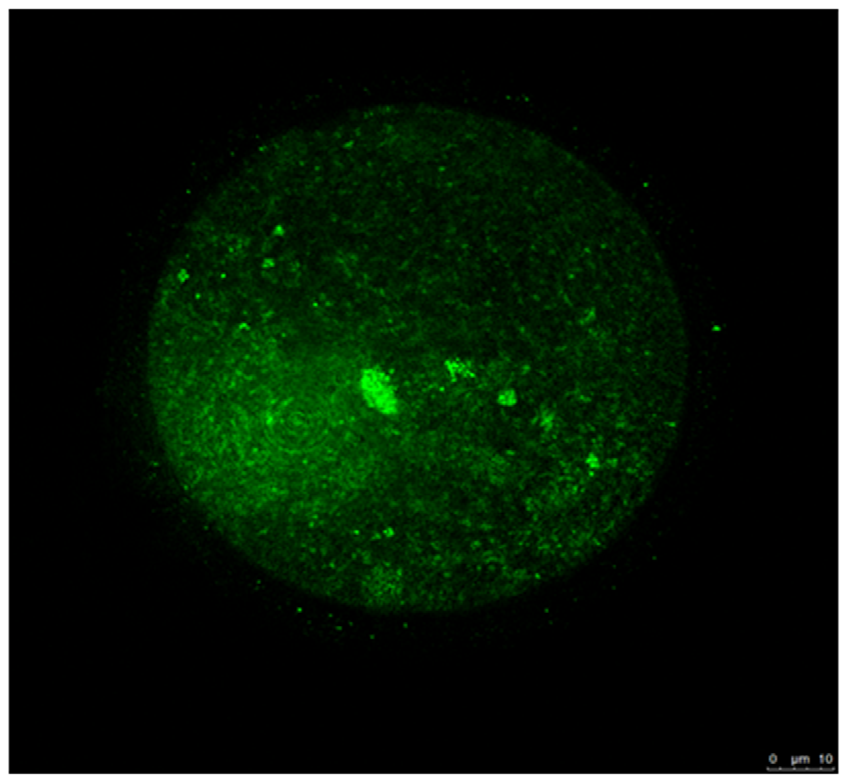
The kinetics of COOH-PLL in mouse oocytes. Mouse oocytes were exposed to PB1(-) supplement with FITC-labeled COOH-PLL (5% (w/v)). Green: FITC-labeled COOH-PLL.

**Figure 2:**
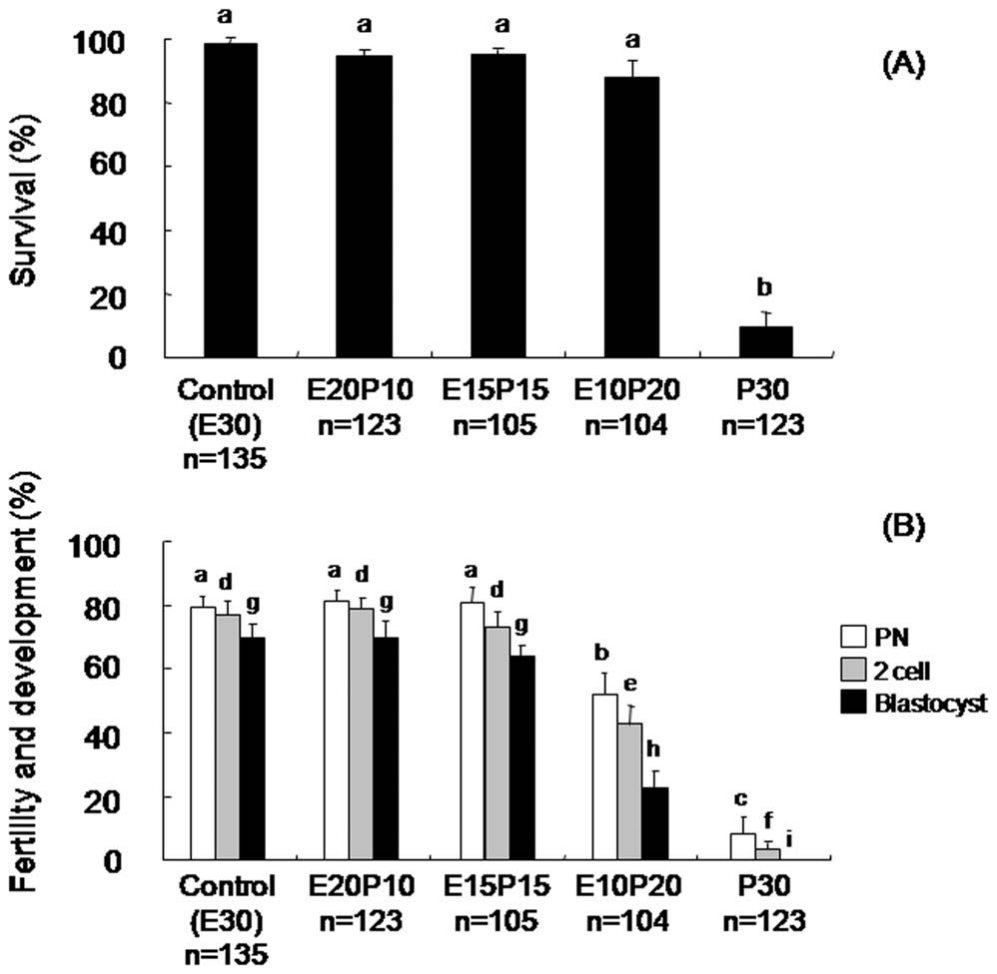
The effects of COOH-PLL on survival, fertility and developmental ability of vitrified mouse oocytes after IVF. Data are shown as means ± S.E.M. Different superscripts denote a significant difference (P<0.05). Numbers of oocytes used in each group were described under the treatment group.

To clarify the characteristics of COOH-PLL, we exposed oocytes to the solution supplemented with FITC-tagged COOH-PLL. These oocytes were examined under a fluorescence microscope. As shown in Figure 1, fluorescence intensity to some extent was observed in the oocyte cytoplasm. The survival rates of the control, E20P10, E15P15, and E10P20 groups were high (99.0±1.2%, 94.6±1.4%, 94.8±2.2%, and 88.2±5.3%, respectively) (Fig. 2A). The oocytes vitrified with COOH-PLL alone as a CPA (the P30 group) showed a low survival rate (9.7±4.5%). The pronuclear (PN) formation rates of the E20P10 and E15P15 groups were also high (81.4±4.9% and 80.8±6.8%, respectively) and were equivalent to that of the control group (79.6±3.7%) (Fig. 2B). By contrast, the E10P20 and P30 groups showed significantly low PN formation rates (52.1±6.2% and 8.2±5.7%, respectively). The rates of the 2-cell stage in the E20P10 and E15P15 groups were also high (78.8±5.8% and 73.6±5.8%, respectively) and equivalent to that of the control group (77.0±4.7%). On the other hand, the rates of the E10P20 and P30 groups were significantly lower (42.9±5.2% and 3.4±2.0%, respectively) than those of the other groups. As for the development to the blastocyst stage, the rates were high in the E20P10 (70.2±7.8%) and E15P15 (64.0±3.8%) groups, and these were equivalent to that of the control group (69.9±4.8%). The rate of the E20P10 group was low (22.7±5.1%). None of the oocytes developed to blastocysts in the P30 group.

We next examined the developmental ability of the oocytes after IVF to term. These results are shown in Table 2. In the control group, 92 embryos were transferred to six recipients and 32 pups were obtained (34.8±2.7%). In the E20P10 group, the rate of pups was significantly improved (37 of 80 embryos, 46.2±3.1%). In the E15P15 group, 72 embryos were transferred to five recipients and 12 pups were obtained (16.7±2.5%). The rate was significantly lower than that of the control and E20P10 groups. In all groups, the pups were visually normal.

**Table 2:** In vivo development of mouse oocytes vitrified with COOH-PLL.

Oocytes	Transferred embryos	Pregnant/recipients (%)	Offspring (%)
Control (E30)	92	6/6 (100)	32 (34.8±2.7)^a^
E20P10	80	5/5 (100)	37 (46.2±3.1)^b^
E15P15	72	5/5 (100)	12 (16.7±2.5)^c^

## Discussion

The successful cryopreservation of oocytes is highly desirable because it leads to a high efficiency of oocyte cryopreservation, making large numbers of viable oocytes available for the generation of offspring via IVF and intracytoplasmic sperm injection. If the oocytes can be efficiently cryopreserved, the cryopreservation protocol will be useful for the high-speed production of not only gene-modified mice but also hybrid mice from different gene-modified mice [20], [21]. Our previous study demonstrated that calcium-free and EG-supplemented media dramatically improved the fertility and developmental ability of vitrified mouse oocytes after IVF in ICR mice [4] and C57BL/6J mice [15]. Here we examined whether COOH-PLL, which was recently developed as a new CPA, is useful for the further improvement of oocyte vitrification.

In the present study, in vitro developmental ability of oocytes vitrified with combined solution with EG and COOH-PLL (E20P10 and E15P15 groups) was high and it was equivalent to that of control (E30 group). Our previous report demonstrated that there was no difference between EG30% group and control (fresh oocytes) [4]. Therefore, the developmental rate even in E20P10 group seems to reach a plateau. In our preliminary study, we tried to use the vitrification media composed of EG30% and PLL10% or 20%. However, we could not improve the developmental ability any more compared to E30 or E20P10 (data not shown). On the other hand, in our present study, in vivo development of vitrified oocytes in E20P10 group was significantly improved, which suggests using COOH-PLL or reducing EG may moderate the invisible damage by vitrification. This may contribute to the increase of in vivo developmental ability beyond the blastocyst stage.

Adversely, oocytes vitrified with COOH-PLL alone showed low survival and developmental ability after IVF. It seems that COOH-PLL has low permeability, because most of the FITC-tagged COOH-PLL was not observed inside the oocyte cytoplasm. Results from vitrification of chondrocyte sheets suggest COOH-PLL is non-permeable CPA [22]. It is unclear how COOH-PLL is involved in the improvement of oocyte vitrified with EG. One possible explanation is that COOH-PLL moderates a rapid increase of osmolality cause by membrane permeable CPAs such as EG although further studies will be required. Therefore, combined media of COOH-PLL with other CPAs which have high permeability is effective for oocyte vitrification. Our data clearly showed that combined treatment with 20% EG and 10% COOH-PLL dramatically improved the mouse offspring compared to that of our previous protocol (E30). One of the advantages of COOH-PLL is its low toxicity because they compared the viabilities of L929 cells cryopreserved with some common CPAs such as dimethyl sulfoxide, glycerol, propylene glycol, polyethylene glycol, and COOH-PLL, and they found that the concentration of PLL up to 20% in the freezing solution did not show detrimental effect on cell viability [10]. Therefore, even in germ cells, using COOH-PLL reduced cytotoxic effect at the cryopreservation of them.

Another advantage of using COOH-PLL is that it is easier than the previously used protocol [4], [15], since in our protocol the cumulus-attached oocytes are vitrified. At the MII stage, cumulus cells are already expanded (Figure S1A), and it requires great skill to handle and vitrify the oocytes before putting these oocytes on the Cryotop. However, COCs exposed in the media supplemented with COOH-PLL showed compacted cumulus cells such as immature COCs (Figure S1B). It is unclear the detailed mechanism why COOH-PLL induced compacted cumulus cells. We speculate that COOH-PLL directly or indirectly affect extracellular matrix of COCs. As a result, it is easier to handle COCs in the media supplemented with COOH-PLL compared to those in the media supplemented with EG alone.

Taken together, our present findings demonstrate for the first time that COOH-PLL is an effective CPA for oocyte vitrification in mice. Although further investigation is required, oocyte vitrification using COOH-PLL seems to be effective for not only mice but also other species including humans.

## Supporting Information

Figure S1 **Morphology of COCs exposed to vitrification media supplemented with EG alone and EG plus COOH-PLL.**
(TIF)
