# Supplementary figures and images for: Efficient Production of Live Offspring from Mouse Oocytes Vitrified with a Novel Cryoprotective Agent, Carboxylated ε-poly-L-lysine
Source: PLoS One. 2013 Dec 23;8(12):e83613. doi: 10.1371/journal.pone.0083613 (PMC3871522; doi:10.1371/journal.pone.0083613)

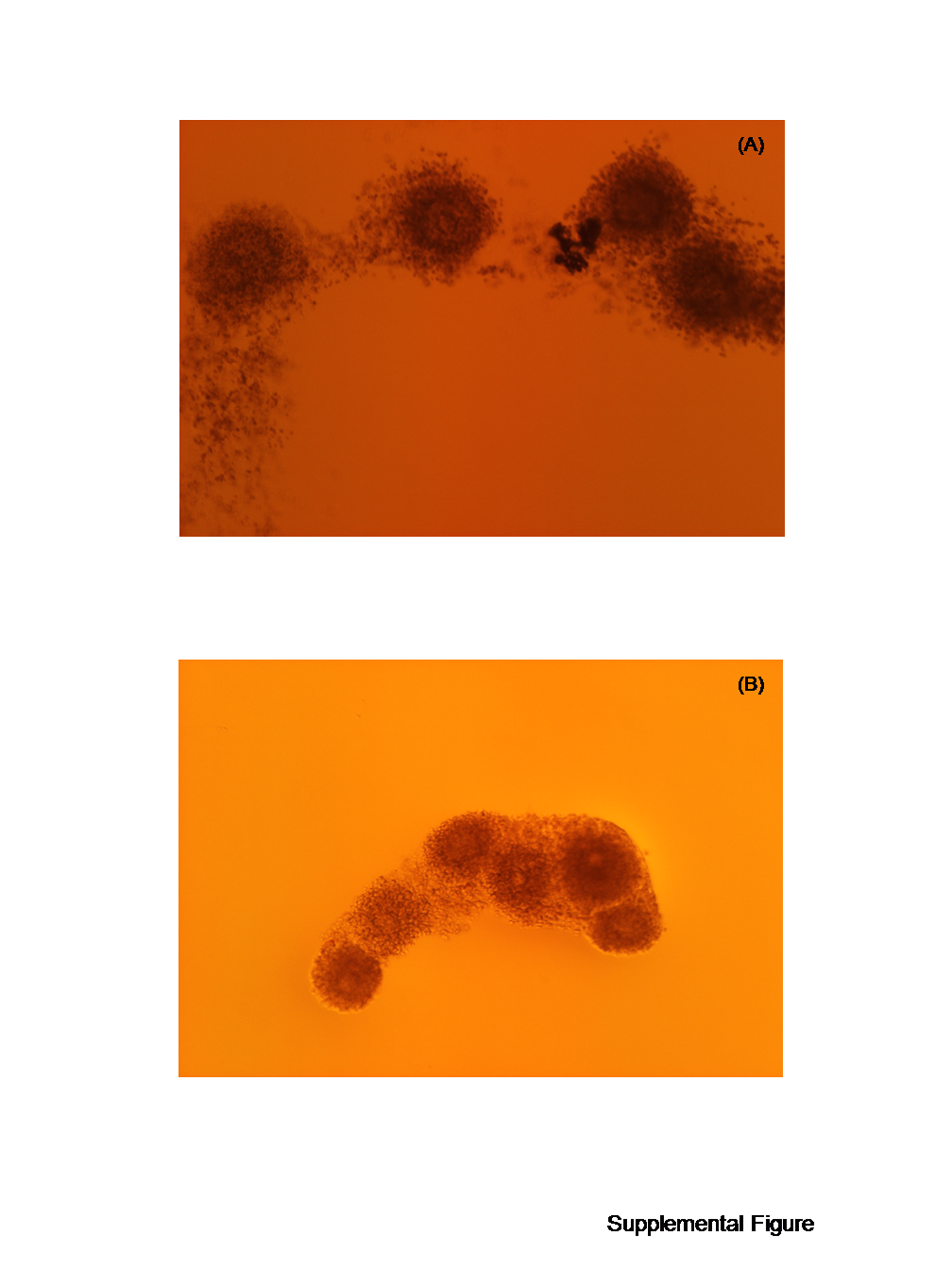

Supplement: Figure S1 — Morphology of COCs exposed to vitrification media supplemented with EG alone and EG plus COOH-PLL. (TIF) [file pone.0083613.s001.tif]
